# Editorial: The model of Ramadan diurnal intermittent fasting: Unraveling the health implications - volume I

**DOI:** 10.3389/fnut.2022.971610

**Published:** 2022-07-19

**Authors:** MoezAlIslam Ezzat Faris, Ismail Laher, Meghit Boumediene Khaled, Ayse L. Mindikoglu, Hassane Zouhal

**Affiliations:** ^1^Department of Clinical Nutrition and Dietetics, University of Sharjah, Sharjah, United Arab Emirates; ^2^Department of Anesthesiology, Pharmacology and Therapeutics, Faculty of Medicine, The University of British Columbia, Vancouver, BC, Canada; ^3^Department of Biology, University of Sidi-Bel-Abbès, Sidi Bel Abbès, Algeria; ^4^Section of Gastroenterology and Hepatology, Margaret M. and Albert B. Alkek Department of Medicine and Division of Abdominal Transplantation, Michael E. DeBakey Department of Surgery, Baylor College of Medicine, Houston, TX, United States; ^5^Department of Science and Techniques of Physical and Sporting Activities (STAPS), University Rennes, M2S (Laboratoire Mouvement, Sport, Santé) – EA 1274, F-35000 Rennes, and Institut International des Sciences du Sport (2I2S), Rennes, France

**Keywords:** Ramadan, intermittent fasting (IF), time-restricted diet, caloric restriction, religious fasting

## Introduction

Intermittent fasting (IF) is rapidly gaining interest across various scientific disciplines and the general community. The practice of IF is a safe and costless intervention that offers health benefits and disease prevention, particularly related to chronic metabolic and aging diseases (1, 2). One of the most commonly practiced models of IF is the obligatory IF observed annually in the month of Ramadan by about 1.5 billion Muslim people. Much evidence gathered during the last two decades suggests that observing this 1-month religious fasting, lasting between 10 and 21 h a day depending on the location and season (3), provides metabolic and physiological benefits.

The many health benefits of Ramadan diurnal intermittent fasting (RDIF) include improvements in body weight and body composition (4–6), reducing complications of the metabolic syndrome (4), improving lipid profiles and other cardiometabolic risk factors (7–10). Further, in healthy people, RDIF helps improve glucose homeostasis (11), ameliorate inflammatory and oxidative stress markers (10), improve liver function tests (3), and modulate gene expression of various components of the anti-inflammatory and antioxidant defense and circadian rhythm systems (12, 13). In subjects with metabolic syndrome, RDIF was shown to improve several components of metabolic syndrome, upregulate tumor-suppressor proteins and downregulate tumor-promoter proteins (14). Despite being the most extensively studied form of IF, many gaps remain in our understanding of the many effects of RDIF in healthy people, including athletes. Further, it is unclear how the observance of RDIF affects patients with illnesses such as diabetes, cardiovascular disease, and cancer. More information is needed on the effects of RDIF on different body systems and the possible genetic expressions and epigenetic changes produced by this religious practice. A better understanding of RDIF will help optimize the practice of RDIF, maximize its health benefits, and guide healthcare providers to better advise their chronically ill patients on matters related to RDIF. Accordingly, variable aspects of RDIF are summarized in this Frontiers Research Topic “*The Model of Ramadan Diurnal Intermittent Fasting: Unraveling the Health Implications—Volume I*.” This Research Topic aims to provide a comprehensive view of studies on RDIF, using observational and interventional studies in humans and animals.

### Summary of selected articles from this Research Topic

About 530 proposed contributors were invited to participate in this Research Topic, from whom we received 14 abstracts and 26 manuscripts. After vigorous screening and a critical review, 15 articles were selected for this Research Topic. The contributing 80 authors were from 16 countries across five continents, including Lebanon, the United Arab Emirates (UAE), the United States of America, Germany, Tunisia, Italy, Canada, Jordan, United Kingdom, Libya, Australia, Singapore, Algeria, Netherlands, China, and Iran. This Research Topic received more than 33,400 views and downloads as of June 2022.

The study by Shatila et al. characterized food intake among Lebanese adults observant of RDIF and compared it to their intake for the remainder of the year. In a year-round observational study, the authors observed significant increases in dietary intakes for 12 out of 19 food groups such as intakes of cereals, cereal-based products, pasta, eggs, nuts and seeds, milk and dairy, and fats and oils were lower, while vegetables, dried fruit, Arabic sweets, cakes and pastries, and sugar-sweetened-beverages intakes during Ramadan. Such differences in the intakes of food groups were also reflected in nutrient intakes, where intakes of carbohydrates, cholesterol, calcium, beta-carotene, vitamin C, folate, and magnesium showed significant changes. This interesting study highlights important differences in dietary food groups and nutrient intakes during the fasting month compared to the rest of the year.

The authors of Riat et al. examined the association between mood-related symptoms and health-related quality of life and several biological parameters, including serum cortisol, brain-derived neurotrophic factor (BNDF), insulin growth factor-1 (IGF-1), interleukin (IL)-8, matrix metalloproteinase (MMP)-9 and myoglobin levels in 34 healthy adult subjects who practiced RDIF. They showed that the cortisol levels were significantly lower 1 week after RDIF compared with the levels measured 1 week before RDIF (*P* < 0.001), and BDNF levels were significantly lower during the last days of RDIF compared with the levels measured 1 week before RDIF (*P* < 0.05). The authors concluded that the effects of RDIF on mood-related symptoms were correlated with different biological parameters, specifically cortisol and BDNF levels.

The authors of Fekih et al. evaluated the effects of mental training through imagery on the competitive anxiety of adolescent tennis players fasting during Ramadan. They studied 38 male tennis players that were randomly allocated to the experimental group (EG) and control group (CG); the CG watched historical videos of the Olympics, while those in the EG performed mental training. There was a significant interaction for all competitive anxiety subscales, with higher intensity and direction scores for cognitive and somatic anxiety subscales during Ramadan for both groups. Higher intensity and direction scores for the cognitive and somatic anxiety subscales occurred during Ramadan for both groups; this increase in scores was greater for the control group than for the EG during the middle and at the end of Ramadan (*P* < 0.001). Intensity and direction scores were significantly lower during Ramadan for the two groups. Further, the score for the intensity of self-confidence was greater for the EG compared with the CG. The authors concluded that mental imagery training reduced cognitive and somatic anxiety and increased self-confidence in the intensity dimension of adolescent tennis players who fast during Ramadan.

The study by Al-Nawaiseh et al. investigated the impact of RDIF on runners' performances, using 15 trained male distance runners who observed Ramadan. Each participant reported to the human performance lab before and at the end of Ramadan. The participants performed graded exercise tests on a treadmill, and their VO_2_, heart rate, time to exhaustion, and running speed were recorded. There were no significant effects of Ramadan fasting on body mass, body fat, lean body mass, VO_2_max, energy availability, and protein intake. However, carbohydrate, lipid, water, and caloric intakes were significantly reduced during Ramadan. Daily training duration and exercise energy expenditure were also significantly reduced after Ramadan. Time to exhaustion and maximal running speed was improved, as were time to exhaustion and maximal running speed of the distance runners; these changes were independent of changes in nutrient intake observed during the study. The authors concluded that the performance of distance runners could be maintained or even slightly improved following the month of Ramadan fasting.

The genetic study by Madkour et al. examined RDIF-associated changes in the Fat mass and obesity-associated (*FTO*) relative gene expression in a group of 63 metabolically healthy subjects with overweight and obesity. The expressions of *FTO* were significantly decreased at the end of Ramadan by more than one-third of the pre-fasting gene expression levels (−32.30%, 95% CI−0.052 −0.981). Significant reductions occurred in body weight, BMI, fat mass, body fat percent, hip circumference, LDL, IL-6, TNF-α, and waist circumference, while there were increases in HDL and IL-10 at the end of Ramadan. Binary logistic regression analysis for genetic expressions indicated no significant association between high-energy intake, waist circumference, or obesity and *FTO* gene expression. The authors concluded that RDIF is associated with the downregulation of the *FTO* gene expression in subjects with obesity, which may explain, at least in part, the favorable metabolic effects of RDIF. Thus, RDIF presumably entails a protective effect on body weight gain and its adverse metabolic-related derangements in subjects with obesity.

In this narrative review, Elmajnoun et al. summarized the impact of the COVID-19 pandemic on children and young adults with type 2 diabetes (T2DM). The authors also explored the potential of intermittent fasting in reversing the pathogenesis of diabetes, highlighting how this could prevent these patients from developing chronic complications. The authors concluded that children and young adults with T2DM are not at risk of severe COVID-19 as is the case in adults with diabetes. However, more research is needed to identify the impact of COVID-19 in children and young adults with T2DM, particularly investigating the efficacy and safety of intermittent fasting, including Ramadan fasting. Moreover, the authors advised that implementing these cost-effective programs could greatly minimize diabetes in children and young adults. Furthermore, this could be particularly effective in patients with prediabetes.

Exercise and fasting confer health benefits independently, leading Zainudin et al. to propose that people who are fasting, especially those experiencing health and clinical challenges, continually engage in physical activity during the Ramadan fasting month. In this opinion piece, the authors recommended walking football (WF) as an exercise of choice among Muslims who are fasting. WF can be played by any individual regardless of their fitness level, skills, and age. WF elicits cardiovascular and metabolic stress responses, which can be beneficial in populations with low fitness levels. Most importantly, WF has the inherent characteristics of being a fun team activity requiring social interactions among participants and, hence, likely to encourage long-term consistent and sustainable participation.

The study by Muammar et al. investigated the outcomes of RDIF using multiple daily insulin injections and continuous subcutaneous insulin infusions to assess patterns of glycemic control and severity of complications during RDIF in older children and adolescents with type-1 diabetes mellitus (T1DM). The effects of dose adjustment, health professional teams, and parental support on safety and glycemic outcomes in children and adolescents with T1DM during Ramadan were also investigated. The results indicated no significant deterioration in indicators of overall glycemic control, which remained inadequate during RDIF. The authors concluded that RDIF should be discouraged in children with poorly controlled T1DM.

There are significant changes in sleep-wake patterns during Ramadan, which are largely caused by alterations in the timings of the two daily meals—one pre-dawn (*Sohor*) and the other at sunset (*Iftar*). The literature review by Bencharif et al. discusses the guidelines for drug treatment (Ramadan often requires altering treatment protocols for people with non-communicable diseases such as diabetes), physical exercise, body composition, and metabolic changes. The impact of the Covid-10 pandemic is also reviewed. The authors summarize international guidelines (from 1995 to 2021) that attempt to optimize the management of diabetes during Ramadan.

The authors previously reported that RDIF caused increases in the concentrations of short-chain fatty acids (SCFA) in the gut microbiome of healthy humans that were associated with improved metabolic parameters, an effect that could be due to a combination of psychological effects and microbiome remodeling. The current study by Su et al. examined changes in the gut microbiome in a mouse model of RDIF where the effects of these two variables (psychological effects and microbiome remodeling) could be isolated. The findings in the mouse model of RDIF confirmed the results of microbiome remodeling in humans of increases in bacteria that stimulate the production of SCFA (which are associated with reduced visceral fat mass in humans).

Using flash glucose monitoring (FGM) monitor in 24 patients with type 1 and types 2 diabetes on insulin therapy who were remotely connected to the diabetes clinics in the UAE; Helal et al. tried to examine the impact of COVID-19 lockdown on glycemic control pre- and post-lockdown and during RDIF. Analyses of data were performed on glucose management indicator (GMI), time in range (TIR), time in hyperglycemia, time in hypoglycemia, low blood glucose index (LBGI), and high blood glucose index (HBGI). Variables were calculated for each period: 30 days before lockdown, 30 days into lockdown and pre-Ramadan, and 30 days into lockdown and Ramadan, using the continuous glucose monitoring analysis package in R-studio software. Results revealed that the mean average glucose (MAG) remained steady before and during the lockdown. The significant difference in GMI and percentage of time in hyperglycemia were reported between Ramadan and pre-Ramadan during the lockdown period. The percentage of TIR was significantly lower in Ramadan as compared to pre-Ramadan. Mean absolute glucose (MAG) and HBGI were found to be significantly higher in Ramadan compared to the pre-Ramadan period. The authors concluded that the lockdown period did not significantly impact the markers of glycemic control in the population studied. However, the authors found that several changes were embedded during the Ramadan fasting period, including increased GMI, HBGI, and glycemic variability similar to what has been reported in other Ramadan studies.

In their study using liquid chromatography-mass spectrometry-based metabolomics technique, Chen et al. investigated the composition of fecal metabolites in Chinese and Pakistani individuals before and after RDIF. The distinct separation of metabolite profiles among ethnic groups as well as between pre-and post-fasting samples was performed. After RDIF, the whole population groups showed significant differences in their respective contents of various fecal metabolites, particularly L-histidine, lycofawcine, and cordycepin concentrations, which were higher after RDIF in the Chinese group. However, brucine was enriched in the Pakistani group. The Kyoto Encyclopedia of Genes and Genomes analysis suggested that metabolites related to purine metabolism, 2-oxocarboxylic acid metabolism, and lysine degradation were significantly enriched in the total subject population pre-RDIF vs. post-RDIF comparisons. Several bacterial taxa were found to be significantly correlated with specific metabolites unique to each ethnic group, suggesting that changes in fecal metabolite profiles related to RDIF may be influenced by associated shifts in gut microbiota. The authors concluded that RDIF-related differences in fecal metabolites, together with these group-specific correlations between metabolites and taxa, support their previous findings that ethnic differences in dietary composition drive the variation in gut microbial composition and diversity.

This systematic review analyzed insulin dosing recommendations that can reduce hypoglycemic events and improve glycemic control for patients with T2DM during the fasting month of Ramadan. Kieu et al. evaluated the findings of 14 eligible studies that included 2,969 study participants across four continents with an average age of 54.8 years. The studies consisted of five RCT studies, and nine observational cohort studies, of which six studies examined insulin dosage adjustment on glycemic control and hypoglycemia during Ramadan, three examined newer ultra-long-acting insulins, and another three compared insulin analogs. The systematic review indicates that insulin dose reduction could prevent hypoglycemia without causing subsequent hyperglycemia, and rapid-acting insulin analogs could improve post-iftar and overall blood glucose without incurring hypoglycemia. This systematic review exclusively analyzed insulin subtypes and dosing strategies for insulin-treated T2DM during Ramadan. The authors recommend more research to confirm the benefits of ultra-long-acting insulins as well as the use of flexible glycemic targets and recommend more randomized controlled trials out before more detailed conclusions could be made.

The authors Mousavi et al. used systematic reviews to summarize current findings on the impact of RDIF and non-RDIF on the gut microbiome. Several databases were used to identify 28 studies (of human and animal model) for this systematic review. The results indicate a significant shift in the gut microbiome, especially in increases of *Lactobacillus* and *Bifidobacteria* following fasting diets. Some studies reported increases in bacterial diversity and production of beneficial metabolites such as SCFA and decreases in inflammatory processes. However, other investigations reported adverse effects of fasting diets on the structure of the microbiome. The authors conclude that most animal and human investigations indicate positive effects of fasting on the composition and structure of the gut microbiome.

The impact of RDIF on the salivary flow rate (SFR) and metabolic parameters was addressed in this systematic review by Besbes et al.. After reviewing the PubMed database, six (06) original articles meeting the inclusion criteria were included in the systematic review. Several parameters were considered in this systematic review which displayed downward trends: SFR was decreased by 10%, the circadian pattern of melatonin remained constant, while melatonin levels, glucose, uric acid, and aspartate aminotransferase (AST) decreased. Levels of alkaline phosphatase (ALP) level increased significantly. The cortisol concentrations in saliva remain unchanged or increased during the fasting days of Ramadan. Salivary levels of immunoglobulin A (IgA) decreased during the last week of the Ramadan fasting month. The authors of this systematic review provided some recommendations for the design of future research studies based on the limitations of some studies e.g., different methodologies used to examine the impact of RDIF on SFR.

## Conclusion

This Frontiers Research Topic contributes to our understanding of the impact of RDIF on various aspects of human nutrition, health, and disease. This Research Topic will hopefully open a venue for further studies and stimulate future discussions among researchers.

## Author contributions

All authors listed have made a substantial, direct, and intellectual contribution to the work and approved it for publication.

## Conflict of interest

The authors declare that the research was conducted in the absence of any commercial or financial relationships that could be construed as a potential conflict of interest.

## Publisher's note

All claims expressed in this article are solely those of the authors and do not necessarily represent those of their affiliated organizations, or those of the publisher, the editors and the reviewers. Any product that may be evaluated in this article, or claim that may be made by its manufacturer, is not guaranteed or endorsed by the publisher.
